# Academy of Stomatology

**Published:** 1904-06

**Authors:** 

**Affiliations:** Academy of Stomatology


### ACADEMY OF STOMATOLOGY.

THE regular meeting of the Academy of Stomatology of Philadelphia was held on the evening of Tuesday, December 22, 1903, at the rooms of the Academy, 1731 Chestnut Street, the President, Dr. L. Foster Jack, in the chair.

A paper was read by Dr. M. H. Cryer, entitled "Impacted Teeth: Their Diagnosis, Liberation, and Extraction," and illustrated by the use of lantern slides.

(For Dr. Cryer's paper, see page 321.)

DISCUSSION.

*Dr. E. T. Darby.*—If we were to study carefully, in this relation, every mouth we look into, we would probably find few people who have thirty-two teeth fully erupted. Out of the six or eight patients I saw to-day, but one had the full complement. One woman thirty years of age had never had any third molars.

It has been said that the size of the human jaw has been deteriorating for hundreds of years. I remember hearing, some twenty-five or thirty years ago, in our State Society, a paper by Dr. Welcher, in which he took the ground that the wisdom-tooth was unnecessary, and that in time there would be no wisdom-teeth in the mouth. When in Egypt, over thirty years ago, I examined hundreds of ancient skulls, but did not find a single jaw which was not large enough for thirty-two teeth. I do not remember seeing any with the wisdom-teeth missing where the subject had been old enough to have them. If it be true that the jaw is deteriorating in size, that may account for the present frequency of impaction of wisdom-teeth.

In the case of a young woman of twenty-six or thirty, seen in my practice, who never had had a lateral incisor, there was a bulging just under the lip near the nose. Suspecting an impacted tooth, I made an incision, which revealed the central incisor twisted around the lateral in a perfect hook. The question arises as to what was the cause of this.

The fact that so many inferior wisdom-teeth are tipped forward, with the occlusal surface abutting against the distal surface of the second molar, raises the question as to whether the tooth can be extracted without injury and a great deal of trouble. I have for years been in the habit of sending such cases to Dr. Thomas. He has often advised the removal of the second molar, hoping that the third molar might right itself and take a normal position; but, experience proves that this tooth rarely becomes useful. In such a case a perfectly sound second molar has been lost and a tooth that is of little value has been saved. When I find a molar tipping forward in that way, with its occlusal surface against the distal surface of a second molar, I prefer not to risk the possibility of fracture at the ramus; on the contrary, I much prefer to cut down with a large bur, exposing the anterior portion of the ramus, and take out the tooth through the opening thus made.

*Dr. F. C. Van Woert,* Brooklyn.—I am here mainly in the interests of the X-ray, being thoroughly convinced that when these obscure cases are presented to us, as general practitioners we should refer them to specialists in that particular line. I cannot quite agree with Dr. Cryer in some of his deductions regarding the X-ray, and yet I fully realize how difficult it is to diagnose some of these skiagraphs.

I have brought over some slides and shall be pleased to show them and tell you some of the difficulties of making a diagnosis from them. The skiagraph does not always present a clear definition owing to the superimposition of one shadow upon another. One requires at times some clinical knowledge of the case itself, otherwise the readings from the skiagraph may possibly be misleading. In such a case as this, which was referred to me by Dr. S. G. Perry, you can plainly see that there are two permanent laterals. The crowns were equally good, and upon the possible condition of the roots depended the extraction of one of the teeth for orthodontic purposes, as you see the root of the lateral next the one to be moved, and which would naturally be extracted, is better than that of the other lateral. Upon the valuable evidence of the skiagraph the distal lateral was removed.

In some of these other cases the evidence is not so clear. An interesting discussion was precipitated during the exhibit between Drs. Van Woert and Cryer, the latter claiming that the location of an impacted lower molar could not be determined by a skiagraph, whereas Dr. Van Woert held that the intensity of the shadows of the alveolar process would give a fairly approximate idea as to whether a tooth lay to the buccal or the lingual side of the lower jaw, while the outline of the tooth would give its other position.

*Dr. M. K. Kassabian.*—Dentistry and medicine have derived equal benefits from the introduction and use of the X-rays. I will confine my remarks to one class of dental anomalies for the treatment of which this discovery can be utilized,—*i.e.*, unerupted teeth. Many difficult cases have been brought to my attention by the dental profession at large, and especially those which have been kindly referred to by Dr. Cryer. Most of Dr. Cryer's cases were those of an obscure nature, and with great difficulty and skilful use of the explorer he diagnosed the conditions which I confirmed by using the Röntgen rays, thus showing and proving the existing conditions by skiagraphs. The ordinary method of probing with an explorer did not always locate the absent tooth, especially in some cases of bicuspids and third molars. These had insinuated themselves deeply beneath other teeth, and the alveolar process was so dense that nothing but the X-ray would locate and determine their position.

An invaluable return from using the X-ray in these dental cases is the exact and precise results obtained. It not only locates the unerupted tooth, but also determines the position in which it is resting. For the benefit of those who are not conversant with X-ray work, I will describe how the location and position of a tooth can be best gotten.

Two skiagraphs are taken from two angles or directions, to determine whether we have a buccal, lingual, distal, or mesial presentment, and I might mention here that Dr. Cryer suggested that I make stereoscopic skiagrams, which permit viewing by a reflecting stereoscope, and as a result, instead of observing flat pictures, we obtain a relief or stereoscopic perspective effect. I have received very good results with my stereoscopic skiagrams at the Philadelphia Hospital, where I use a special table which I had built for that purpose. I employ two methods for skiagraphing dental conditions,—A, the intra-oral method; B, the extra-oral or buccal method.

A. The intra-oral method is proceeded with by inserting a small piece of film (which is light and moisture-proof) over the gum tissue at a point where trouble is suspected, placing the tube in such a position that perpendicular rays will be cast upon the teeth and film. This method covers a smaller area, but produces a picture with very sharp detail, and is especially recommended for anterior teeth.

B. The extra-oral or buccal method requires that a plate 5 x 7 be brought in contact with the jaw at the region of suspected trouble. The patient is directed to incline the neck and head to an angle measuring about forty-five degrees. The tube is now placed over the shoulder on the opposite side at a distance of twenty inches from the face, to avoid superimposition of the jaws. This process produces a picture of great area, and is intended for bicuspids and molars.

During exposure of this plate it is wise to insert a block of wood about two inches square between the teeth, which prevents the patient from closing the mouth. These methods produce pictures which to the novice appear difficult to intelligently read, but, as in other lines of our professional work, only time and experience teach and give masterly results.

The slides exhibited here this evening show the great progress that has been made in this line of work and how much assistance is rendered in diagnosing unerupted teeth by the aid of the X-ray.

*Dr. Pfahler.*—As Dr. Cryer has told you, I am not a dentist, but as a doctor of medicine I have done some X-ray work. Just as Dr. Cryer has learned to use his probe most skilfully, so in X-ray work we must learn to use the X-ray skilfully; and in order to obtain results we must do two things,—make good negatives, and know how to read them. The mistakes which seemed to be so prominent to-night were, I think, no fault of the X-ray, but rather of the man who read them. This subject is still in its infancy. Some one asked why the shadow of the loose piece of bone lying in a pus-cavity was darker than the surrounding bone. If you will look at that carefully you will find that the shadow was not darker than the surrounding bone, but it stood out more prominently, because of the light area surrounding it. The difficulty Dr. Cryer has mentioned in the case of an impacted tooth is not one that cannot be overcome. Their exact location can be determined by the method which Dr. Van Woert has shown you, and again brought out by Dr. Kassabian. You can determine the proximity of a foreign body to the plate by the sharpness of the shadows and by their overlapping. Another method is by fluoroscopic study. This is not yet thoroughly worked out, but will be in a short time. By these various methods we locate foreign bodies, and can probably come within a millimetre of the exact location of one of these teeth. All these difficulties can be overcome as our experience increases.

*Dr. Cryer* (closing).—Dr. Darby asked about the cause of impaction. I tried to make that plain in my paper. If we study the anatomical structure of normal bone of the jaw, the lower for instance, we find that the cancellated portion slides within the cortical portion from the back forward. This sliding is accomplished as the teeth are developed. It has been demonstrated that the development of the teeth changes their relation to the mental foramen until the latter, instead of being at the base of the cuspid tooth, comes to be between the two bicuspids, and in some cases under the first molar. This sliding motion allows the teeth to move into their proper position. If this motion is interfered with we have impaction. The occurrence of an inflammatory condition in the cancellated bone near the cortical will cause the osteoblasts to build bone at that point, which will lock them together. The teeth being developed in little bony capsules, these will be cemented to the cortical portion of the bone, which does not move. It appears to me, that this is the reason of impaction. To avoid this condition, we must constantly keep the mouths of our children in perfect health, and allow nothing to exist which will cause a productive inflammation.

In answer to Dr. Van Woert, I do not consider the X-ray of little use to us. It is bound to be of great use to us, but, as Dr. Pfahler has said, we must learn to read the pictures. The pictures which Dr. Van Woert has shown prove conclusively to my mind that he cannot tell what he sees in these pictures. In one case he said he could not tell whether it was bone until it was removed. The most important use of the X-ray in this connection will be in the region of the canine, lateral, and central teeth.

Otto E. Inglis,
*Editor Academy of Stomatology.*

